# Nest attendance by tropical and temperate passerine birds: Same constancy, different strategy

**DOI:** 10.1002/ece3.5812

**Published:** 2019-11-21

**Authors:** Suzanne H. Austin, William Douglas Robinson, Vincenzo A. Ellis, Tara Rodden Robinson, Robert E. Ricklefs

**Affiliations:** ^1^ Department of Fisheries and Wildlife Oregon State University Corvallis OR USA; ^2^ Department of Biology University of Missouri‐St Louis St. Louis MO USA; ^3^ Department of Biology Lund University Lund Sweden; ^4^ Department of Entomology and Wildlife Ecology University of Delaware Newark DE USA

**Keywords:** incubation, latitudinal gradient, life‐history evolution, parental care, passerine birds

## Abstract

1. Parental care in birds varies among species and geographic regions. Incubation behavior influences embryonic development rate and varies substantially among species.
2. We studied attendance at the nest by videoing nests or collecting data from the literature for 112 species in north temperate and lowland tropical sites, then associated patterns of incubation on‐ and off‐bouts with species and environmental traits.
3. Songbirds nesting at low elevations incubate their eggs for an average of 74.1% (±12.9 *SD*, *n* = 60 species) of the time in temperate regions and 71.0% (±12.2 *SD*, *n* = 52 species) in tropical regions during daylight hours, and 84.3% (±8.2 *SD*) and 85.3% (±6.2 *SD*), respectively, of each 24‐hr cycle.
4. While these attendance percentages do not differ significantly between latitudes, our data also show that lowland tropical songbirds make fewer visits to the nest and, consequently, have longer on‐bouts and off‐bouts during incubation. This pattern in attendance reflects a latitudinal contrast in parental care strategy, where lowland tropical birds reduce visits to the nest by increasing on‐ and off‐bout lengths while maintaining the same proportion of time spent incubating their eggs (constancy).
5. Similar constancy across latitude suggests that tropical and temperate birds may be similarly constrained to maintain elevated egg temperatures for normal embryo growth.
6. The different attendance strategies adopted in each region may reflect differences in ambient temperature, adult foraging time, and nest predation rate. Consistently warm ambient temperatures likely allow tropical birds to take longer off‐bouts, and thereby to reduce activity around the nest, compared to temperate birds.

## Graphical abstract

We compared avian attendance data during incubation of 112 New World temperate and tropical passerine species. The amount of time that parents incubated their eggs did not differ between regions, but tropical birds employed a different attendance strategy. Tropical parents spent longer periods of time on and off of the eggs, made fewer trips to the nest, but incubated for the same percentage of time.

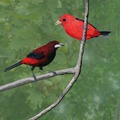

## 1 INTRODUCTION

Life‐history theory suggests that suites of traits covary in relation to parental investment in reproduction and survival (Roff, 1992). The resulting covariation in traits among species produces pace‐of‐life syndromes such that species with similar strategies occupy similar positions along a slow–fast continuum (Ricklefs & Wikelski, 2002). In birds, egg‐laying combined with contact incubation challenges parents to optimize investment of time and energy between caring for their eggs and self‐maintenance. At the fast end of the pace‐of‐life continuum, species tend to have higher basal metabolic rates, more numerous offspring, shorter development periods, faster growth, and higher rates of offspring provisioning, consistent with greater allocation of resources to reproduction compared to self‐maintenance (Martin, 2008; Martin, Auer, Bassar, Niklison, & Lloyd, 2007; Martin, Ton, & Niklison, 2013; Moreau, 1944; Ricklefs, 1969b, 1976, 1993, 2000; Skutch, 1962; Tieleman et al., 2006; Tieleman, Williams, & Ricklefs, 2004; Wiersma, Muñoz‐Garcia, Walker, & Williams, 2007). Such species dominate temperate environments, where annual adult survival is low, on average, compared to tropical environments (Karr, Nichols, Klimkiewicz, & Brawn, 1990; Ricklefs, 1997; Ricklefs & Shea, 2007; Sandercock, Beissinger, & Stoleson, 2000). In contrast, many tropical species have lower basal metabolic rates, smaller clutches, longer incubation periods, slower postnatal growth, and lower rates of nestling provisioning, which places them toward the slow end of the pace‐of‐life continuum (Tieleman et al., 2006; Wiersma et al., 2007; Williams, Miller, Harper, & Wiersma, 2010).

Differences in the duration of incubation between otherwise similar birds on the fast and slow ends of the life‐history axis have puzzled biologists. Two main hypotheses have been proposed. One suggests that high levels of nest predation in the tropics cause parents to reduce activity around the nest, thereby reducing risk to themselves and their offspring. A consequence of this strategy is reduced average incubation (egg) temperatures and reduced rates of food provisioning to the offspring, resulting in slow offspring growth and development (Chalfoun & Martin, 2007; Martin, 2002; Robinson & Rompré, 2008; Skutch, 1949, 1985). Alternatively, slow growth and development, particularly of the embryo, for which resources are provided in the egg at laying, might be associated with increased quality of the offspring. One hypothesis states that slow growth is associated with increased resistance to pathogens by the immune system, which may increase reproductive success and extend adult life span (Ricklefs, 1992; Ricklefs, Austin, & Robinson, 2017). However, in a small comparative study of 10–12 temperate species, Palacios and Martin (2006) either found no correlation between incubation period blood parasite prevalence or a negative correlation between incubation and phytohaemagglutinin (PHA) of nestlings.

Key requirements for embryo development include heat (maintenance of embryos above a physiological zero temperature for efficient growth), humidity (sufficient to keep water loss over incubation below 10%–15% of egg mass), and, in many species, movement (to assist in respiration and nutrient movement within the egg and to prevent adhesion of the embryo to shell membranes) (reviewed in Deeming, 2002). Temperature plays a clear role in embryonic development, and higher temperature can accelerate growth within certain bounds. However, how much of the embryonic development period is determined by external versus internal constraints remains unanswered. For instance, does parental attendance behavior, as opposed to intrinsic differences in embryo development rates, drive observed variation in incubation periods? One hypothesis suggests that more frequent adult activity at the nest might increase the risk that visual predators will detect and depredate nests (Robinson, Rompré, & Robinson, 2005; Skutch, 1949, 1985). Adult activity includes parents traveling to and from their nests to attend eggs or to feed young. Because tropical birds experience, on average, higher nest predation rates than temperate species (Brawn et al., 2011; Libsch et al., 2008; Ricklefs, 1969a; Robinson, Robinson, Robinson, & Brawn, 2000), decreasing nest attendance might allow tropical birds to reduce already high rates of nest discovery by predators (Martin, 2002, 2015; Martin, Scott, & Menge, 2000). However, a consequence of reduced nest attendance is that eggs may cool sufficiently to extend the length of the embryo developmental period (Martin et al., 2013).

Evidence from both observational and experimental studies indicates that incubation periods are prolonged when eggs are left unattended. Greater constancy of nest attendance (on‐bouts) keeps egg temperatures higher overall and shortens incubation periods (Martin et al., 2007; Ricklefs, 1987). Lower nest attendance is usually with a consequence of prolonged off‐bouts (Skutch, 1962), which can result in egg temperatures falling below the level required for optimal embryonic development (Olson, Vleck, & Vleck, 2006). Hatching time decreases with increasing incubation temperature, but extreme deviations from optimal incubation temperature (ca. 37.5°C) also can cause embryo deformities and mortality, and reduce the fitness of hatchlings (Christensen, 1995; Feast, Noble, Speake, & Ferguson, 1998; Landauer, 1967; Romanoff, 1935). For example, deviations from optimal incubation temperatures, whether high or low, at different times during the incubation period affect skeletal growth in poultry (Yalcin, Özkan, & Siegel, 2012). Studies at high‐altitude tropical sites have shown that the lower ambient temperatures combined with lower nest attendance lead to lower egg temperature and reduced embryo metabolism, which result in longer incubation periods (Martin et al., 2007, 2013). Skutch (1962) associated lower attendance with insectivory (requiring more time for capturing food items) in suboscine birds and, more generally, with conspicuousness of the nest. He also suggested that the lower bound of nest attendance was set by requirements of the embryo, while the upper bound was set by the availability of food to the parents.

Although the relationship between nest attendance, egg temperature, and incubation period seems intuitive, recent work has provided mixed evidence in wild birds. Egg swapping experiments have shown that eggs of species normally incubated at lower temperatures have shorter incubation periods when incubated in nests of species that maintain their eggs at higher temperatures (Martin et al., 2007). In contrast, Robinson and colleagues (Robinson, Austin, Robinson, & Ricklefs, 2013; Robinson, Styrsky, Payne, Harper, & Thompson, 2008) utilized artificial incubators to maintain eggs of tropical house wrens (*Troglodytes aedon inquietus*), temperate house wrens (*T. a. aedon*), and several lowland tropical songbirds at constant high temperature (i.e., 36.5°C), which removed the effects of parental attendance behavior on incubation period. Neither treatment consistently reduced the duration of incubation. Nor did constant incubation at the same temperature reduce variation among species in the length of the embryo development period. Two correlational studies (Ricklefs, 1993; Tieleman et al., 2004) found no relationship between incubation temperature and nest attendance or incubation period.

Variation in embryo developmental rate might also be related, at least partly, to differences among species in mechanisms to reduce posthatching mortality (Ricklefs, 1992; Ricklefs et al., 2017; Robinson et al., 2013, 2008; Tieleman et al., 2004). Embryo development and adult incubation behavior presumably reflect selection on both to maximize individual fitness in a given environment (Tinbergen & Williams, 2002; Vleck & Vleck, 1996; Williams, 1996). Ricklefs (1992) and colleagues (Ricklefs, Ellis, Medeiros, & Svensson‐Coelho, 2018) found a negative correlation across species between the prevalence of malaria in an avian population and the length of the species' embryo development period. This pattern suggested that slower embryo development might be associated with increased effectiveness of the immune system in reducing the health consequences of malaria infection. DNA breakage and lipid peroxidation levels in embryos also were lower in longer‐lived species with longer incubation periods (Tsunekage, 2013; Tsunekage & Ricklefs, 2015). Thus, slower growing embryos appear to suffer less oxidative damage than those that grow rapidly.

**Figure 1:**
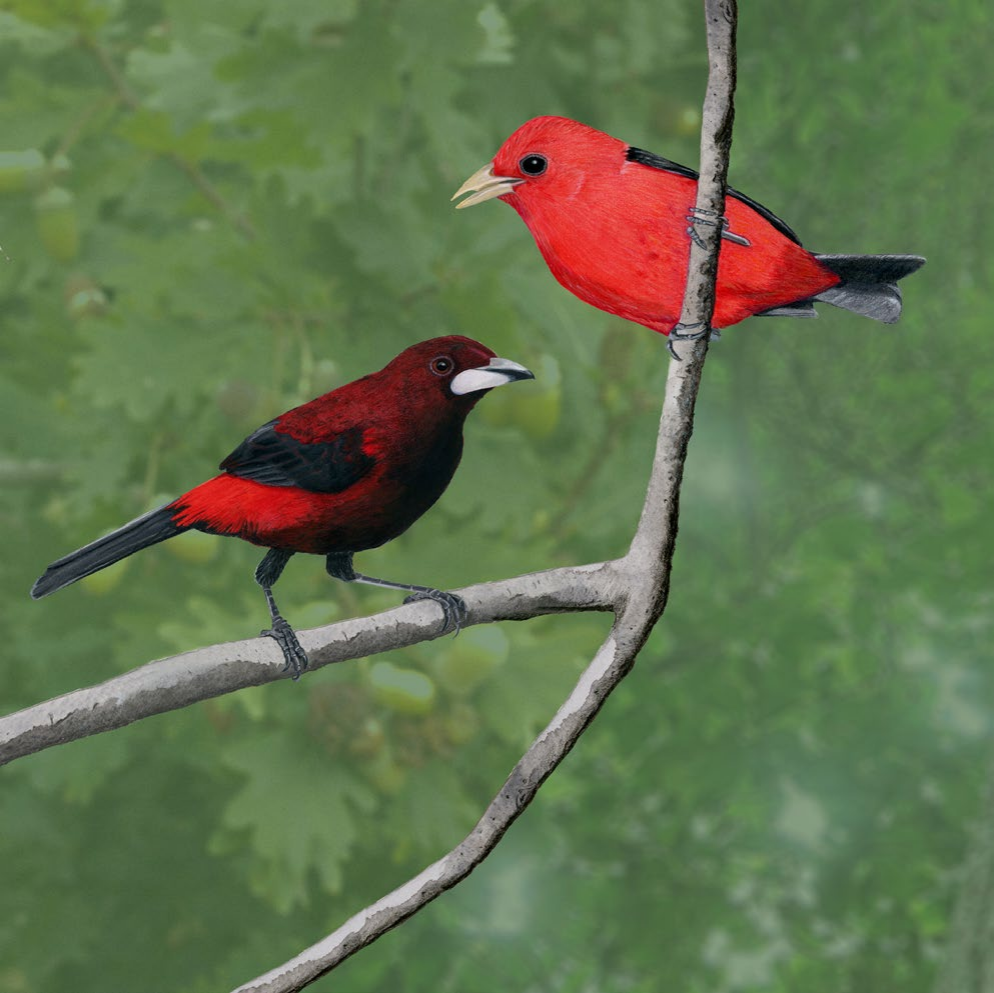
An illustration of the temperate scarlet tanager (*Piranga olivacea*) and tropical crimson‐backed tanager (*Ramphocelus dimidiatus*) by Tara Rodden Robinson

Here, we quantify variation in nest attendance, in relation to other life‐history traits, across a diverse sample of 112 tropical and temperate songbird species based on original data and the literature (Figure 1). Our objectives were to determine whether nest attendance in north temperate species exceeds that in lowland tropical species, as might be expected from the cooler environmental temperatures but overall shorter incubation periods in temperate regions. We also assessed how nest attendance covaried with other life‐history traits to create integrated reproductive strategies among our sample of birds.

## 2 MATERIAL AND METHODS

### 2.1 Study areas

We conducted research at a temperate site (Michigan, USA) and a tropical site (Republic of Panama). In southwestern Michigan (42N 85W), we worked at the Lux Arbor Reserve and the Kellogg Biological Station (KBS). Lux Arbor is a 1323‐ha reserve that consists of agricultural fields, mixed‐deciduous forest, conifer tree plantations, wetlands, and meadows (http://lter.kbs.msu.edu/). Average annual precipitation is 89 cm, and average annual temperature is 9.7°C. KBS is approximately 17‐km south of Lux Arbor and consists of landscaped parkland on the grounds of the biological station. Data collection occurred between May and early August from 2003 to 2005. In central Panama (9N 79W), we conducted fieldwork in Soberania National Park, approximately 30‐km north of Panama City near the junction of the Chagres River and the Panama Canal. We worked primarily in and around the small town of Gamboa in lowland second‐growth rainforest and parkland habitats. Average rainfall is 260 cm/year (Robinson et al., 2000), and average annual temperature is 25°C (Windsor, 1990). Fieldwork was conducted between March and July annually from 2003 to 2006.

### 2.2 Data collection

At each site, we measured life‐history and natural‐history traits of nesting birds. We monitored nests every 3 days until failure or fledging of young, with the exception of dates near nesting‐stage transitions (e.g., nest completion, laying, hatching) when we checked nests daily in order to estimate incubation and nestling periods. Due to the taxonomic breadth of this study, when values were missing or sample sizes were low, we supplemented our data with literature values or used literature values alone. For more information on original data, see Austin (2013).

#### 2.2.1 Nest attendance variables

To quantify nest attendance and adult activity, we videotaped nests for 2‐hr intervals using Sony Hi8 handycams. We deployed cameras near nests at all times of day but focused on the first 8 hr after sunrise. We measured constanc*y* (defined as the percentage of time parents attended the nest during an observation period), on‐bout length (the duration of a single period of adult attendance), off‐bout length (the time adult(s) were absent from the nest between on‐bouts, and thus, the nest was unattended), and visit rate (the number of visits adults made to their nest per unit of time). Because some birds may have been affected by our initial visit to their nest to place the camera, we began each observation period when a parent first returned to the nest after this disturbance.

Following onset of incubation and clutch completion, adults typically incubate their eggs through the night. To estimate total nest constancy for each species, we combined our daytime measurement of attendance with nighttime attendance (100% attendance at night in both temperate and tropical locations, which we confirmed with nocturnal nest inspections and videos; see also Ricklefs & Brawn, 2013). We estimated diel (total) constancy as the proportion of time on the nest during the 24‐hr cycle: Constancy_total_ = [(Constancy_day_ * Day length, hr) + (Constancy_night_ * Night length, hr)]/24 hr. During our study periods, days in Panama were shorter (12.12 hr) and nights were longer (11.88 hr), on average, compared to Michigan (15.07 and 8.93 hr, respectively).

#### 2.2.2 Egg mass and clutch size

We measured egg mass (g) and quantified clutch size as the number of eggs per clutch. We individually marked each egg with a nontoxic felt‐tipped pen, weighed (±0.1 g) each egg, and measured its length and breadth (±0.1 mm). If eggs were found after incubation had commenced, we used the linear measurements of individual eggs rather than our measured masses to estimate mass because eggs lose 12%–15% of their mass over the course of incubation. The formula is: *W* = 5.48 × 10^‐4^ L * *B* ^2^, where *W* is the estimated fresh egg mass (g), *L* is the length of the egg (mm), and *B* is the breadth of the egg (mm) (Deeming, 2002). We also used this formula for literature data unless fresh egg mass was noted.

#### 2.2.3 Incubation period

We measured incubation period as the duration of time (day) from clutch completion to hatch of the last egg.

#### 2.2.4 Adult mass

Adult mass (g) for each species was obtained from Wiersma et al. (2007), Hau, Ricklefs, Wikelski, Lee, and Brawn (2010), or Dunning (2008). For species exhibiting sexual size dimorphism, we averaged male and female masses.

#### 2.2.5 Daily Nest Mortality Rate (DMR)

Daily Nest Mortality Rate was calculated using the method of Mayfield (1961, 1975) for species with samples of *n* ≥ 15 nests. For species with <15 nests, we calculated DMR using (Ricklefs, 1969a): DMR = −ln(*S*)/*t*, where *S* is proportion of nests that survived to fledging and *t* is the length of time that nests had contents. We pooled data within study sites across years to generate one DMR estimate per species and site. For species with smaller samples of nests, we supplemented our data with values from the literature.

#### 2.2.6 Categorical variables

We determined each species' incubation strategy (uniparental vs. biparental) and nest type (open cup, enclosed cup, and cavity/burrow) from our observations and from the literature. A variable describing geographic location (i.e., region: north temperate vs. lowland tropical) also was included in our analyses.

#### 2.2.7 Summary statistics of continuous traits

We used mean or median values of traits for comparisons among species (PROC UNIVARIATE; SAS 9.3). We evaluated the normality of distributions with Shapiro–Wilk tests. If the Shapiro–Wilk test indicated significant (*p* < .05) divergence from normality, or if the sample was ≤4 nests, we used median values rather than means; otherwise, we used means.

### 2.3 Statistical methods

All continuous traits were scaled by 0.01 and log‐transformed prior to analysis to improve the distributions of the variables; off‐bout length was scaled by adding 1 min prior to log‐transformation to account for the value of 0 for the species *Ramphocaenus melanurus*, which has continuous incubation. Nest height was square‐root‐transformed to improve the distribution. All conventional analyses were conducted in SAS (v.9.3).

#### 2.3.1 Regional comparison

We compared all attendance traits to region (PROC MIXED). Least squares means are computed by subtracting the parameter estimates for the levels of a categorical variable of interest (as with contrasts). We note the direction of the pair‐wise comparison differences in the text. For all comparisons, we present back‐transformed estimates (*e* ^estimate^), and interpret magnitude differences between the variables. Thus, an exponentiated estimate of *e* ^0^ = 1.00 would indicate a 1.0‐fold relationship, or 0% difference, between the regions while *e* ^0.6575^ = 1.93 would indicate a 1.93‐fold relationship, or 93% difference. Standard error terms remain in ln‐transformed units because back‐transformation would provide spurious results as it assumes that values are symmetrical around the mean. To account for the phylogenetic structure of the data, all linear models were also run in a phylogenetic generalized least squares (pGLS) framework.

We also conducted an analysis of variance (PROC MIXED) to confirm that daily nest mortality rate (DMR) differed between regions after accounting for nest type because, while tropical regions tend to have higher nest predation rates, nest type also strongly influences nest mortality.

#### 2.3.2 Incubation type comparison

**Figure 2:**
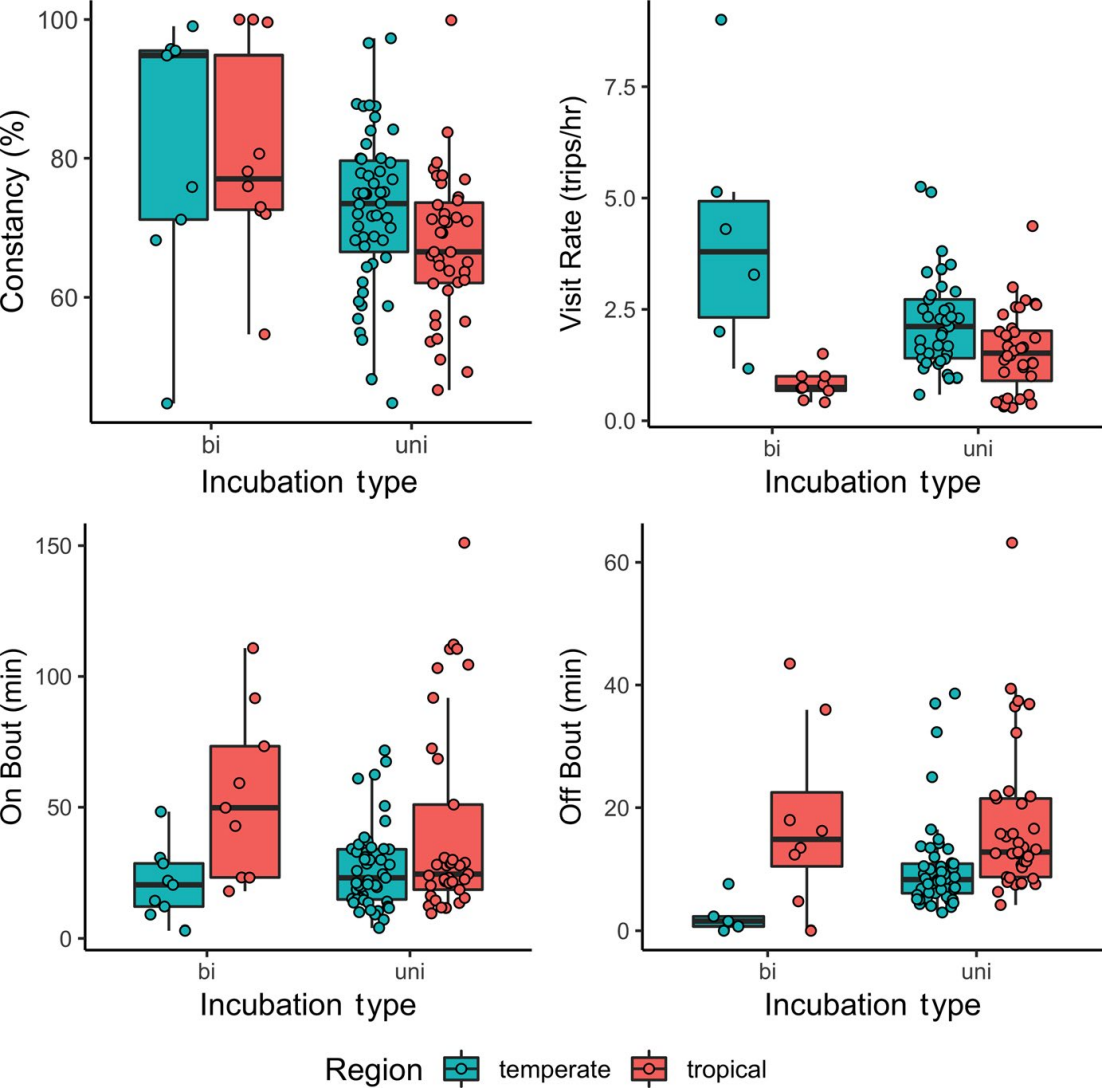
Panel box plot of attendance variables (diurnal constancy, off‐bout, on‐bout, and visit rate) with overlaid data points by incubation type split by region (aqua = temperate, red = tropical)

We compared daytime and total constancy of nest attendance among incubation types (uniparental vs. biparental), regions (Figure 2). Initially, we added their interaction term, but the interaction term was not significant for either constancy (region: *F*_1, 105_ = 0.9, *p* = .345, incubation type: *F*_1, 105_ = 11.0, *p *= .001 region*incubation type: *F*_1, 105_ = 0.6, *p* = .450) or total constancy (region: *F*_1, 105_ = 0.9, *p* = .335, incubation type: *F*_1, 105_ = 9.5, *p* = .003, region * incubation type: *F*_1, 105_ = 0.2, *p* = .642); thus, we present the analyses without this term. It should be noted that due to the relative rarity of species that engage in uniparental incubation in our dataset (*n* = 19; temperate = 9, tropical = 10) and the phylogenetic bias (i.e., all but one uniparentally incubating species in our tropical samples was a suboscine whereas all species in our temperate sample were oscines). When variables other than constancy were considered, samples sizes ranged from 13 to 15 (temperate = 5–9 and tropical = 8–10). Thus, we subset our dataset to include species with only uniparental incubation (the predominant type in both regions).

#### 2.3.3 Principal Components Analysis (PCA)

We conducted a PCA (PROC FACTOR) to assess how attendance traits covaried with region and identified independent axes of variation of these interrelated variables.

#### 2.3.4 Model selection and averaging

We assessed how each attendance variable was related to key natural‐ and life‐history variables among a subset of 60 species for which we had complete data for our traits of interest. Our focal traits (i.e., *y*‐variables) included constancy, on‐bout length, off‐bout length, and visit rate (number of parental visits to the nest, hr^‐1^), while our life‐history and natural‐history traits (i.e., *x*‐variables) were adult mass, egg mass, region, clutch size, incubation period, incubation type, nest type, and DMR. Several variables were multicollinear (adult mass vs. egg mass, *r* = 0.94; clutch size vs. region, *r* = 0.78). To avoid variance inflation, we excluded redundant variables from the models, but we interpreted these traits (egg mass and region) through their collinear variables (adult mass and clutch size). For each model, we conducted bootstrapping and model averaging to determine the variables that occurred most frequently and had the most weight (PROC GLMSELECT, ModelAverage; Burnham & Anderson, 2002). We report weight (*π_i_*) of the top model based on the model weight from bootstrapped models. Using the same approach, we also conducted region‐specific model selection to determine if and how variables varied independent of region.

### 2.4 Phylogenetic analyses

For these analyses, we downloaded 2,500 phylogenetic trees from http://birdtree.org (Jetz, Thomas, Joy, Hartmann, & Mooers, 2012), “Stage2_MayrAll_Hackett”) and used them to create a maximum clade credibility tree in TreeAnnotator (Drummond, Suchard, Xie, & Rambaut, 2012), which we then trimmed to include only the species in our analysis. The phylogenies at http://birdtree.org do not include the subspecies *T. aedon inquietus*, so we manually added that species to the final tree. We did this by using the function “bind.tip” in the R package phytools (Revell, 2011) to add a node connecting *T. aedon aedon* and *T. aedon inquietus* at 2 mya; the two subspecies show 4% divergence at cytochrome *b* (Mann, Barker, Graves, Dingess‐Mann, & Slater, 2006). We used the resulting tree to optimize Pagel's lambda (a measure of phylogenetic signal) (Freckleton, Harvey, & Pagel, 2002) for the residuals of each model and to estimate model parameters following Revell (Revell, 2010). These analyses used the gls() function from the package nlme (Pinheiro, Bates, DebRoy, Sarker, & R Core Team, 2017) and the corPagel() function from the package ape (Paradis, Claude, & Strimmer, 2004) in R v.3.4.0 (R Core Team, 2017). In three of the standard models, the optimizer failed to converge on an estimate for Pagel's lambda. For two of those models (visit rate by region for uniparental incubating species only and rate of off‐bouts by region for uniparental incubating species only), the log likelihoods of the models increased as lambda approached zero suggesting little or no phylogenetic effect to correct for. For the third model (rate of off‐bouts by region for all species), the log likelihood of the model increased and remained relatively constant for values of lambda less than about 0.75. For these three models, we do not report pGLS results. For all other models, we present the *p*‐values from the pGLS models following the conventional results. We also ran pGLS on the models determined by model averaging, however, the optimizer only converged on an estimate for Pagel's lambda for one model (constancy as a function of nest type, DMR, clutch size, and nest height); the log likelihoods of the other models all increased as lambda approached zero. Similar issues were present when we compared region‐specific models; thus, we only reported results from models that converged.

## 3 RESULTS

### 3.1 Regional comparison

Nest attendance did not differ between regions (constancy = 1.05‐fold difference ± 0.03 [temperate‐tropical], *F*_1,110_ = 2.3, *p* = .13, pGLS *p* = .29; total constancy = 0.98 ± 0.02, *F*_1, 111_ = 1.7, *p* = .20; pGLS *p* = .20). In contrast, visit rates were significantly lower (1.93 ± 0.13, *F*_1, 89_ = 25.5, *p* < .001, pGLS *p* < .001), and on‐ and off‐bouts were significantly longer (on: 0.63 ± 0.13, *F*_1,105_ = 11.8, *p* < .001, pGLS *p* = .002; off: 0.57 ± 0.13, *F*_1,100_ = 18.4, *p* < .001), in tropical birds.

We found that DMR was higher in the tropics (1.27‐fold difference ± *SE* =0.12, *F*_1,101_ = 3.8, *p* = .054, pGLS *p* = .057; nest type *F*_2, 101_ = 17.5, *p* < .001, pGLS *p* < .001).

### 3.2 Incubation type comparison

We found that constancy was significantly related to incubation type (1.2‐fold difference [bi/uni] ± 0.04, *F*_1,106_ = 11.0, *p* = .001, pGLS *p* = .006), but not region (1.06 ± 0.03, F_1,106_ = 3.6, *p* = .062; pGLS *p* = .20; Figure 2). Biparental incubators tend to produce higher total constancy than species with uniparental incubation. Comparisons of total 24‐hr constancy across regions paralleled those for daytime constancy (Region: 0.99 ± 0.02, *F*_1, 106_ = 0.8, *p* = .38, pGLS *p* = .17; Incubation Type = 1.07 ± 0.02, *F*_1, 106_ = 9.5, *p* = .003, pGLS *p* = .039). Among uniparental incubating species, we found no statistically significant difference in constancy between regions after phylogeny was accounted for (constancy: *F*_1, 88_ = 4.8, *p* = .032, pGLS *p* = .16; total constancy: *F*_1, 88_ = 0.4, *p* = .51, pGLS *p* = .25; Table 1). Recall that *constancy* constitutes percent daytime incubation, whereas *total constancy* is an estimate of diel constancy (constancy_day_ scaled by each region's average photoperiod and assuming 100% constancy at night). Visit rates, on‐bout lengths, and off‐bout lengths of uniparental incubators showed similar trends as reported in the regional comparisons (visit rate: *F*_1, 71_ = 10.4, *p* = .002; on‐bout: *F*_1, 84_ = 4.1, *p* = .045, pGLS *p* = .029; off‐bout: *F*_1, 84_ = 16.3, *p* < .001; Figure 2; Table 1). We also determined whether bi‐parental incubating species varied across region. We found similar patterns to uniparental species—both constancy variables did not vary by region and visit rate, on‐bout and off‐bout did (Table 1). Unfortunately, we could not control for phylogeny among bi‐parental incubating species because it was confounded by region: All except one species in the tropical biparental dataset were suboscine passerines while all temperate species were oscines.

**Table 1:** Regional variation in constancy, total constancy, visit rate, on‐bout, and off‐bout among uniparental and bi‐parental incubating passerines

Variable	Uniparental				Bi‐parental		
	*df*	*F*	*p*	pGLS *p*	*df*	*F*	*p*
Constancy	1, 88	4.8	.032	0.166	1, 17	0.0	.935
Total constancy	1, 88	0.4	.51	0.246	1, 17	0.4	.564
Visit rate	1, 71	10.4	.002	–	1, 13	31.4	<.001
On‐bout	1, 84	4.1	.045	0.029	1, 16	9.7	.007
Off‐bout	1, 84	16.3	<.001	–	1, 11	7.8	.018

Note

pGLS *p*‐values did not converge for either uniparental visit rate or off‐bout length and all bi‐parental comparisons and were not reported.

### 3.3 Principal Components Analysis (PCA)

Associations between attendance traits revealed that region, visit rate, on‐bout length, and off‐bout length explained much of the variance (55%) in the PCA (PC1, Table 2). The second axis (28%) was associated with constancy, while the third axis (12%) distinguished region with respect to other variables.

**Table 2:** PCA results for attendance variables and region (*n* = 88)

Trait	PC1	PC2	PC3	PC4
Region	−0.60	0.49	0.63	−0.06
Constancy	0.19	0.94	−0.21	0.21
Visit rate	−0.95	−0.13	−0.07	0.21
On‐bout	0.91	0.34	0.06	−0.10
Off‐bout	0.80	−0.38	0.37	0.27
Cumulative variance	0.55	0.83	0.95	0.98

Note

Highlighted factors indicate loadings ≥ 0.50. Region: temperate = 1, tropical = 0.

### 3.4 Model averaging

#### 3.4.1 Constancy

The best explanatory model for constancy included nest type, DMR, clutch size, and nest height (*π_i_* = 1609; nest type *F*_2, 54_ = 6.9, *p* = .002, pGLS *p* = .024 [cup vs. cavity, Dunnett‐adjusted *p* = .001, pGLS *p* = .007; enclosed vs. cavity, *p* = .181, pGLS *p* = .073], DMR *F*_1, 54_ = 4.2, *p* = .046, pGLS *p* = .927, est. = 1.07 ± 0.03; clutch size *F*_1,54_ = 9.5, *p* = .003, pGLS *p* = .260, est. = 1.22 ± 0.07; nest height *F*_1, 54_ = 5.8, *p* = .020, pGLS *p* = .002, est. = 0.00 ± 0.03). Constancy was positively related to DMR, clutch size (redundant with region), and nest height, although DMR and clutch size/region were not correlated with constancy after accounting for phylogeny. Birds with cup nests had 25% (95% CI = 1.09–1.44) higher constancy than cavity‐nesting species. Species with enclosed nests did not significantly differ in constancy from cavity or open‐cup nesting species after accounting for all other variables (est. = 1.14, 95% CI = 0.95–1.35).

#### 3.4.2 On‐bout length

On‐bout length was positively related to incubation period, DMR, and adult mass (*π_i_* = 3,624; incubation period *F*_1,56_ = 11.6, *p* = .001, est. = 5.33 ± 0.49; DMR *F*_1,56_ = 29.8, *p* < .001, est. = 1.79 ± 0.11; adult mass *F*_1,56_ = 13.4, *p* < .001, est. = 1.51 ± 0.11).

#### 3.4.3 Off‐bout length

The best model included incubation period, DMR, adult/egg mass, nest type, incubation type, clutch size/region and nest height. Specifically, off‐bout length was positively related to incubation period, DMR, and adult/egg mass (*π_i_* = 1,243; incubation period *F*_1, 51_ = 7.1, *p* = .010, est. = 2.90 ± 0.40; DMR *F*_1, 51_ = 3.5, *p* = .069, est. = 1.17 ± 0.09; adult mass *F*_1,51_ = 2.7, *p* = .109, est. = 1.14 ± 0.08). Off‐bout length was negatively related to clutch size/region and nest height (clutch size *F*_1, 51_ = 7.1, *p* = .011, est. = 0.62 ± 0.18; nest height *F*_1, 51_ = 11.3, *p* = .002, est. = 0.06 ± 0.07). Of the categorical variables, species with uniparental care had longer off‐bouts, periods where the nest was unattended by the adult(s), than those with biparental care (incubation type *F*_1, 51_ = 13.6, *p* < .001 (uni versus bi = 1.80 ± 0.16, *p* < .001)), while birds with open‐cup nests had significantly shorter off‐bout lengths compared to enclosed‐ and cavity‐nesting species (nest type *F*_2, 51_ = 10.7, *p* < .001 **(**cavity versus cup est. = 1.63 ± 0.16, adj. *p* = .005; enclosed versus cavity est. = 1.23 ± 0.19, *p* = .300)).

#### 3.4.4 Visit rate

Nest visit rate during incubation was negatively related to incubation period, DMR, and adult/egg mass (*π_i_* = 949.8; incubation period *F*_1, 56_ = 12.5, *p* < .001, est. = 0.22 ± 0.43; DMR *F*_1, 56_ = 19.3, *p* < .001, est. = 0.67 ± 0.09; adult mass *F*_1, 56_ = 5.4 and *p* = .025, est. = 0.80 ± 0.10).

### 3.5 Region‐specific model selection

#### 3.5.1 Constancy

In temperate songbirds, we found that constancy was related to nest type, and positively related to DMR, clutch size, and nest height (*π_i_* = 2,831; nest type *F*_2, 32_ = 12.4, *p* < .001, pGLS *p* = .010; DMR *F*_1,32_ = 8.1, *p* = .008, pGLS *p* = .024; clutch size *F*_1, 32_ = 4.3, *p* = .047, pGLS *p* = .010; nest height *F*_1, 32_ = 20.3, *p* < .001, pGLS *p* < .001). However, in tropical passerines, constancy was only positively correlated to adult mass and negatively correlated with nest height (*π_i_* = 2,016; adult mass *F*_1, 19_ = 5.7, *p* = .027; nest height *F*_1, 19_ = 6.1).

#### 3.5.2 On‐bout length

We found that on‐bout length among temperate species was related to nest type negatively correlated with incubation period and positively related to DMR and adult mass (*π_i_* = 1,323; nest type *F*_2, 32_ = 2.6, *p* = .087; incubation period F_1, 32_ = 6.9, *p* = .013; DMR *F*_1, 32_ = 19.1, *p* < .001; adult mass *F*_1, 32_ = 7.6, *p* = .010), while on‐bout length among tropical songbirds was only positively correlated with DMR (*π_i_* = 1,509; *F*_1, 20_ = 5.9, *p* = .025, pGLS *p* < .001).

#### 3.5.3 Off‐bout length

We found that off‐bout length among temperate species was related to incubation type and nest type (*π_i_* = 916; incubation type *F*_1,33_ = 45.1, *p* < .001, pGLS *p* < .001; nest type *F*_2,33_ = 6.3, *p* = .005, pGLS *p* = .537). There was also a suggestive positive relationship between off‐bout length and adult mass (*π_i_* = 2,227; *F*_1,33_ = 2.7, *p* = .109, pGLS *p* = .871). However, the model with the strongest fit for off‐bout length among tropical species was the intercept‐only model; thus, none of the variables we included in the model adequately explained the variation in off‐bout among tropical species.

#### 3.5.4 Visit rate

Finally, we found that incubation visit rate was negatively related to DMR and adult mass (*π_i_* = 947; DMR *F*_1, 35_ = 9.2, *p* = .005; adult mass *F*_1, 35_ = 9.5, *p* = .004) in temperate species, but was only related to incubation type (*π_i_* = 1,468; *F*_1, 20_ = 7.9, *p* = .011) in tropical species.

## 4 DISCUSSION

Among the species included in our study, we found limited support for a latitudinal difference in constancy of nest attendance (diurnal and total). Only when we limited the dataset to species with uniparental incubation did we find a latitude effect, but this effect disappeared when phylogeny was accounted for and in models with total incubation constancy. Across 112 species from 28 families in the order Passeriformes, constancy was similar across regions (diurnal temperate: *n* = 60 species, median = 75.0%, range = 44.7%–99.0%; tropical *n* = 52 species, median = 71.0, range = 46.7%–100%); total constancy: temperate: 84.3, range = 65.3–99.4, tropical: 85.3, range = 73.1%–100%). Even though constancy did not differ between temperate and tropical latitudes, lowland tropical birds visited nests at a lower hourly rate and spent longer periods on and off the nest compared to temperate species. Based on our multivariate analyses of all attendance variables, we found that region, on‐bout, off‐bout, and visit rate were highly interrelated, while constancy was not related to any other variable. These results suggest that tropical birds differ from temperate birds in the strategy they use to attend their nests, but not in how much time they incubate their eggs.

**Figure 3:**
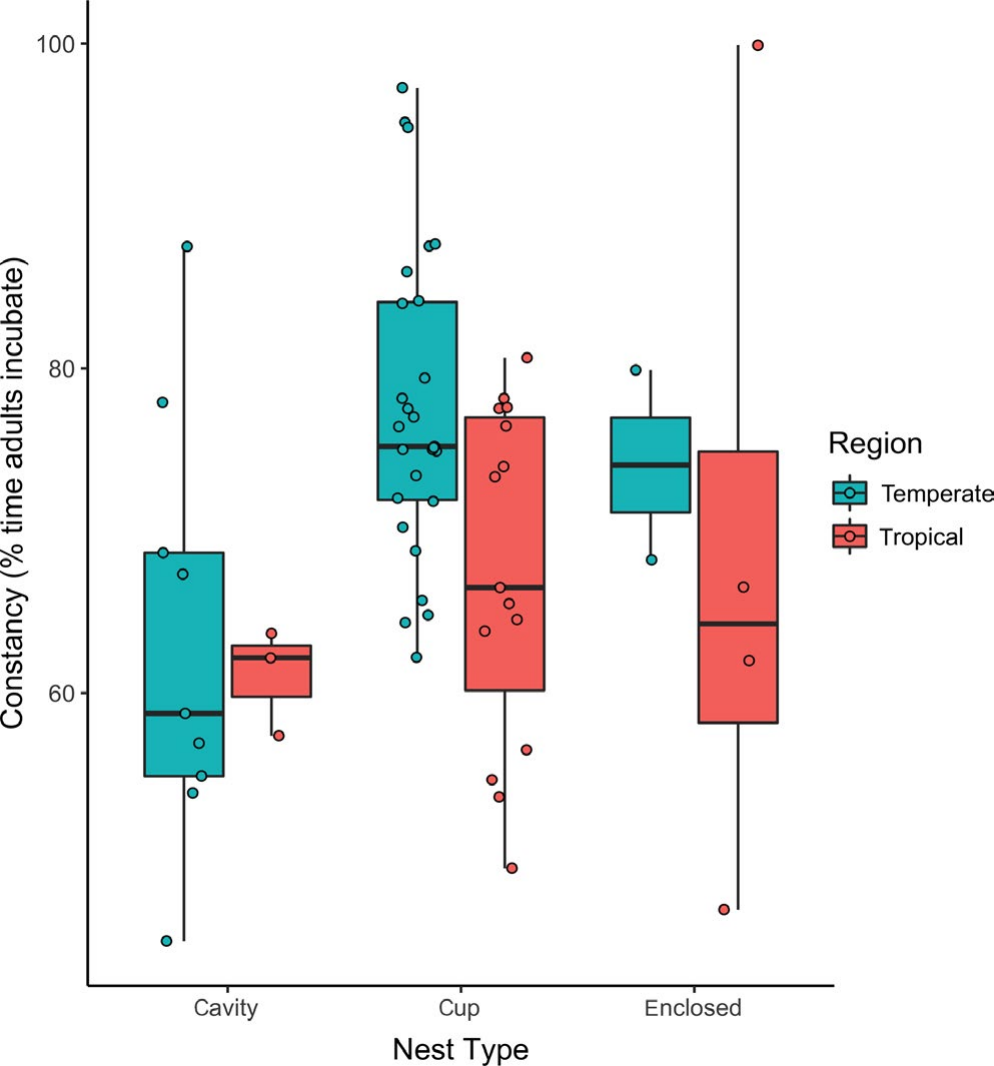
Box plot of diurnal constancy with overlaid data points by nest type split by region (aqua = temperate, red = tropical)

In four linear models of the individual attendance variables (diurnal constancy, on‐bout, off‐bout, and visit rate), we found consistent differences in how these traits correlated with life‐history and natural‐history traits. Constancy was positively related to nest type, DMR, clutch size, and height of the nest from the ground. Many of the *x* (explanatory)‐variables are interrelated (and correlated), which may explain their inclusion within the model. For instance, DMR is associated with nest height, nest type, and clutch size/region. Higher enclosed or cavity nests of temperate species tend to be safer. Species with higher constancy tended to build open‐cup nests, to have higher DMR, and to nest higher off the ground. That open‐cup nesting species tended to have higher constancy than species nesting in cavities (Figure 3) is interesting. Adults incubating in cavity nests are more vulnerable to predators than are open‐cup‐nesting species (reviewed in Ricklefs et al., 2017). Thus, the lower constancy of cavity‐nesting species may be related to adult predation pressure. It could also be related to a more stable microclimate, because cavity nests are typically more sheltered from wind and experience less temperature fluctuation than open‐cup nests. Thermal inertia, associated with larger clutches and larger eggs, could also be playing a role. That is, larger clutches and/or larger eggs are better able to maintain their temperatures following parental departure because additional eggs provide additional insulation from ambient temperatures because less of their surfaces are exposed to ambient temperature. Similarly, larger eggs have a lower surface area to volume ratio than small eggs in smaller clutches; thus, all else being equal, their cooling rate is generally lower than smaller eggs. Because region is not, on its own, correlated with constancy, and clutch size is statistically redundant with region, the relationship between region/clutch size and constancy appears to be caused by the variation in clutch size across nest types. That is, birds with larger clutches tended to nest in cavities, and on average, cavity‐nesting species had lower constancy (65.12%) than enclosed (68.13%) or open‐cup (73.4%) nesting species. Incubation constancy of enclosed‐nesting species did not differ from those using open‐cup or cavity nests, though these results could be attributed to a lack of statistical power; enclosed‐nesting species, and their attendance data, are less common than open‐cup and cavity‐nesting species.

Our statistical model of on‐bout length revealed a positive association with incubation period, DMR, and adult mass. Accordingly, birds with longer on‐bouts also had longer incubation periods, higher nest predation rates, and larger adult/egg masses. Many of these are traits are typically associated with tropical species; however, region was not included in the model suggesting that it did not account for sufficient variation in the model as compared to the retained variables. Again, the complex interrelatedness of variables likely influenced our result, as birds with larger adult/egg masses also tend to have longer incubation periods (Ricklefs & Starck, 1998) and may require longer to reheat their eggs to an optimal temperature following absences from the nest. Off‐bout length was also positively correlated with incubation period, DMR, and adult/egg mass while being negatively related to clutch size/region (longer in the tropics) and nest height. Species with uniparental care had longer off‐bouts than those with biparental care. Species with longer off‐bouts also tended to have smaller clutches, to be tropical, and to nest in cavity or enclosed nests. The inclusion of the clutch size/region variable suggests that this result may simply reflect regional differences. All of the traits included in this model have well‐established latitudinal patterns; thus, the strength of the regional difference in off‐bout may have influenced this result. Nest visit rate was also correlated with incubation period, DMR, and adult mass, though unlike on‐ and off‐bout lengths, the relationships were all negative. Thus, birds that visited their nests at lower rates had higher nest predation rates, larger adult masses, and longer incubation periods.

A predominant hypothesis addressing the latitudinal difference in reproductive strategies suggests that adults minimize attendance to reduce their own risk of mortality at the nest and also not to attract predator attention, thereby improving chances of nests successfully fledging young (i.e., the predation paradox, Martin, 2002). A cost of reducing attendance is potentially increasing the length of the incubation period, which can increase the cumulative mortality risk. The lower nest DMR in temperate habitats suggests that temperate birds should be more constantly attentive (i.e., incubating a higher percentage of time), but free to engage in higher visit rates, due to the lower nest predation pressure. While we found that tropical species, with higher nest predation rates, tend to make fewer visits to the nest per day (using conventional linear models but not pGLS), there is little support for regional differences in constancy, despite differences in DMR. Instead, birds with lower visit rates during incubation have longer on‐ and off‐bouts, but the same overall constancy. Visit rate and off‐bout length follow the pattern expected by the predation paradox (fewer and longer, respectively), but on‐bout length and constancy, thought to be the primary drivers of longer tropical incubation periods, do not (Martin, 2002).

Our findings on constancy support earlier correlational and experimental research showing that predation rate is not related to overall incubation constancy. Our data reveal correlations between DMR and several attendance variables, but the direction of the trends does not support the central idea of the predation paradox—that birds stay away from their nests to reduce predation risk (Martin, 2002). The preponderance of evidence across multiple studies of incubation in lowland tropical birds suggests that innate differences in embryonic development, not predation, are responsible for their longer incubation periods (Ricklefs et al., 2017, 2018; Ricklefs & Brawn, 2013; Robinson et al., 2013, 2008; Tieleman et al., 2004). For instance, Robinson et al. (2013, 2008) found that artificially incubating tropical bird eggs at uniformly high temperatures did not reduce incubation periods, as would be expected if reduced nest attendance caused longer incubation periods in tropical species. Comparative studies have also demonstrated that egg temperatures do not explain regional differences in incubation periods of lowland Panama birds (Tieleman et al., 2004, but see Martin et al., 2007). These studies suggest that egg temperature and constancy are not driving the long incubation periods of tropical birds. Similarly, Ricklefs et al. (2017) showed that longer off‐bouts of tropical species are not a response to the relatively greater risk of nest predation in the tropics.

Higher and more stable ambient temperatures may allow lowland tropical birds to spend longer periods off the nest compared to temperate birds, which encounter more environmental temperature variation during their breeding seasons. In temperate regions, lower ambient temperatures mean that unattended eggs cool faster, which requires birds to return to the nest more frequently to maintain egg temperature (White & Kinney, 1974). Variation in egg temperature with latitude has been attributed to differences in constancy (Schwabl, Palacios, & Martin, 2007, but see, Ricklefs & Brawn, 2013), but our data show that constancy (day and total) is the same, on average, for both regions. Instead, our results suggest that birds of each region use different strategies with respect to bout length. While we did not directly test how ambient temperature interacts with, and influences, adult attendance behavior, we suspect that this has a major influence on selection on this trait.

Birds optimize their attendance based on the needs of the developing embryo and their own self‐maintenance (White & Kinney, 1974). The idea that adults optimize constancy is supported by Chalfoun and Martin (2007), who used food supplements to determine whether food availability or predation rate exerted more influence on adult attendance at the nest. They found that supplemented birds did not increase incubation time, but instead engaged in more self‐maintenance activities (Chalfoun & Martin, 2007). While these authors suggested that high nest predation, not limited food availability, was responsible for the low nest attendance in *Prinia maculosa*, other explanations are possible (Chalfoun & Martin, 2007). In particular, Skutch (1962) suggested that maintenance of embryo temperature sets the lower bound for adult attendance while adult self‐maintenance requirements set the upper bound. When one considers the effects of cooling on the embryo, this becomes a straightforward concept. During long off‐bouts, egg temperature may fall below the range for optimal embryo growth; long periods at suboptimal embryo temperatures halt or retard embryo development, thereby making growth less efficient (Olson, Vleck, & Adams, 2008; Olson et al., 2006). This effect would be more pronounced at lower ambient temperature, where eggs cool faster.

Zebra finch (*Taeniopygia guttata*) embryos exposed to periodic cooling had a lower body mass and used more energy for growth than those incubated under constant temperatures (Olson et al., 2008). Periodic cooling had less effect on linear trait growth than on soft tissue and mass growth, resulting in changes in body proportions (Olson et al., 2008). Embryos incubated at low temperatures also survive less well than those incubated under optimal conditions (Ben‐Ezra & Burness, 2017; Berntsen & Bech, 2015; Durant, Hopkins, Carter, Stachowiak, & Hepp, 2013; Durant, Hopkins, Hawley, & Hepp, 2012; Nilsson, Stjernman, & Nilsson, 2008). Selection should favor a level of incubation constancy that is sufficiently high to avoid such fitness costs to individual embryos, but adults should also seek a strategy that reduces their own fitness costs. In species with low constancy or long off‐bouts, selection should favor embryos that are tolerant of frequent cooling, as in some petrel species (Boersma & Wheelwright, 1979). We doubt that this is an issue for the lowland tropical birds in our study because the lower cooling rate (related to the higher ambient temperatures and high relative humidity in the tropics) probably ameliorates the potential effects of long periods of egg‐neglect.

Similar to other comparative studies, we found no association between constancy and incubation period (Ricklefs, 1993; Tieleman et al., 2004). Normal incubation constancy might balance rapid embryonic development against optimized parental investment. Robinson et al. (2013) and Robinson et al. (2008) provided support for this conclusion. By artificially incubating eggs under constant conditions, these authors removed the effects of parental attendance and of off‐bouts. Contrary to their prediction, removing off‐bouts did not decrease incubation period, meaning that simulated increased constancy did not accelerate embryo development (Robinson et al., 2013, 2008). Ricklefs and Brawn (2013) suggested rate of embryo growth and the length of the incubation period might be, in part, related to intrinsic attributes of the embryo independent of incubation constancy. Tropical embryos and hatchlings may derive benefits from longer incubation periods, including strengthened immune function (Ricklefs, 1992; Ricklefs et al., 2017; Ricklefs & Brawn, 2013). Although we expected that the length of the incubation period would be correlated with incubation constancy (Ricklefs, 1993), we found no such relationship. We did find that that incubation period was related to on‐ and off‐bout length and visit rate (and adult mass). As discussed earlier, longer on‐ and off‐bouts and lower visit rates were associated with larger birds, longer incubation periods, and higher rates of predation.

In conclusion, we found that lowland tropical birds engage in a different strategy, compared to temperate species, for attending their eggs, with longer on‐ and off‐bouts and lower visit rates to the nest. This strategy is unrelated to overall incubation constancy, which did not differ between regions. While DMR seems to influence several attendance traits, the direction runs counter to those hypothesized by the predation paradox. Lowland tropical birds are exposed to higher average DMR but do not reduce overall time at the nest site as expected; rather, they remain longer on the nest during each incubation bout. Longer on‐bouts allow tropical species to incubate their eggs for the same proportion of time as temperate species (constancy and total constancy) despite longer absences from the nest and a lower visit rate. The idea that tropical birds neglect their eggs to reduce risk to themselves is not supported (Boersma & Wheelwright, 1979). We cannot disentangle the effects of ambient temperature and attendance behavior on the length of the incubation period based on these data; however, previous experimental work suggests that constant temperature does not consistently accelerate development. We suggest that the requirements of the embryos interact with environmental conditions, that is, ambient temperature and humidity, to influence the expression of parental care during incubation. Our data and analyses contribute to the growing understanding that lowland tropical species engage in different life‐history strategies than their temperate counterparts. We suggest future work on latitudinal differences of incubation period should focus on intrinsic attributes of the embryo and embryo fitness.

## CONFLICT OF INTEREST

None declared.

## AUTHOR CONTRIBUTIONS

RER and WDR conceived of the project and acquired funding. SHA, TRR, and WDR formulated the experimental design and collected the data. SHA and WDR transcribed the videos. SHA analyzed the data and drafted the manuscript. VAE conducted phylogenetic analyses. SHA, WDR, RER, and VAE wrote the manuscript.

## Data Availability

Austin, S.H., Robinson, W.D., Ellis, V.A., Robinson, T.R., and Ricklefs, R.E. 2019; Attendance Austin_AttendanceAppendix; Dryad; https://doi.org/10.5061/dryad.2c4n774.
